# Climate Change and Drought Events in the Geochemical Records of the Lacustrine Deposits in the Southeastern Tibetan Plateau

**DOI:** 10.1371/journal.pone.0168928

**Published:** 2016-12-29

**Authors:** Wenxiang Zhang, Qingzhong Ming, Zhengtao Shi, Jie Niu, Huai Su

**Affiliations:** 1 Key Laboratory of Geographical Process and Environmental Change in the Plateau of Yunnan Province, Kunming, China; 2 Key Laboratory of Plateau Lake Ecology and Global Change, Yunnan Normal University, Kunming, China, Yunnan University of Finance and Economics, Kunming, China; 3 Yunnan University of Finance and Economics, Kunming, China; Institute of Tibetan Plateau Research Chinese Academy of Sciences, CHINA

## Abstract

Lacustrine deposits at the margin of the southeastern Tibetan Plateau (SETP) are sensitive indicators for the evolution of the southwest Asian monsoon (SWAM) during the Quaternary. Thus, they can provide insight into the Quaternary climatic history and their relationship with global climatic changes. The results of the geochemical analysis of the Xiaozhongdian Basin section at the SETP suggest that SiO_2_ had the highest content of the major elements followed by Al_2_O_3_. The order of the abundance of the major elements was generally as follows: SiO_2_>Al_2_O_3_>Fe_2_O_3_>CaO>MgO>K_2_O>TiO_2_>Na_2_O>MnO_2_. The geochemical proxies, such as chemical index of alteration (CIA), the index of compositional variability (ICV) and (CaO+K_2_O+Na_2_O)/Al_2_O_3_, indicate the weak chemical weathering and the aridification of the margin of the SETP during the Heinrich events. In addition, the aridification of the SETP during the Heinrich events may be closely related to the cold signals transmitted from the high latitudes of the North Atlantic to the TP, and the effect caused the cooling effect to be very strong on the TP as a result of the upper-level westerly jet stream and then reduced the suction action associated with the SWAM, thus accelerating the drying rate of Xiaozhongdian Basin, which was amplifying the degree of drought in Heinrich events.

## Introduction

Geochemical elements are useful indicators of chemical weathering, geological processes and tectonic settings of sedimentary catchments. They can also be used to reconstruct the catchment paleoclimatic and environmental changes [1–3]. Studying the chemical proxies of elements in lacustrine sediments allows us to understand the close interdependence of the Asian monsoon evolution and the global climate changes [4–11]. The Tibetan Plateau (TP) has always been the object of intense climate research [12, 13]. Previous research suggests that the TP is one of the regulators of global climate [14, 15]. The Xiaozhongdian Basin (XB) is located at the margin of the southeastern TP (SETP), the climate is obviously controlled by the Asian southwest monsoon and local climatic influences of the Qinghai-Tibet Plateau. Recent studies have found that historical records and reconstructions showed that variability in summer monsoon precipitation led to droughts [16–18]. And three Quaternary lacustrine deposition cycles and five sand layers have been identified in the XB [19]. Therefore, it is important to provide high-resolution and typically abundant environmental and climatic information to study the impact of the TP on the climatic events of the southwest Asian monsoon (SWAM) region, and it is critical to be able to evaluate the influence of global change on regional climate.

We aim to establish the geochemical weathering processes in the XB and evaluate the coupling mechanism between the Dansgaard-Oescher records of the XB and the TP based on the high-resolution geochemical proxy analysis of XB lacustrine deposits.

## Study Area

**Fig 1:**
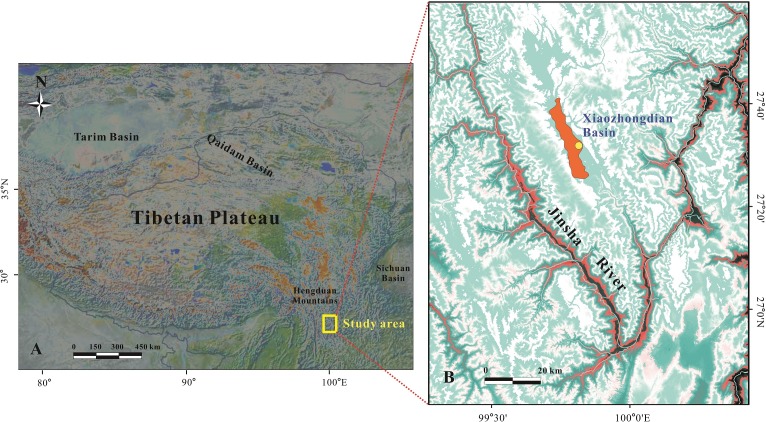
The location and digital elevation model of the Xiaozhongdian Basin lacustrine deposits (the data come from http://srtm.csi.cgiar.org for open-source. The figure is similar but not identical to the original image of SRTM, and is therefore for illustrative purposes only.)

The XB is located in the Hengduan Mountains of the SETP and approximately 80 km northwest of Yulong Mountain (Fig 1). The XB is a Cenozoic faulted depression and it is controlled by the SWAM [20, 21]. Many cycles of lacustrine deposition have occurred in the XB and the total thickness of the deposits in the basin is approximately 100 m over an area of 400 km^2^. The extremely thick lacustrine deposits in the basin have recorded the regional environmental and climatic changes over time; therefore, the XB is an ideal area for studying the evolution of the southwest monsoon. The mean annual temperature for the basin based on data recorded at regional weather stations is approximately 5.8°C and the mean annual precipitation is approximately 850 mm, with more than 85% of the precipitation occurring between June and September [22].

## Sampling and Methods

### Stratigraphy and sampling

A 15.3-m section along the Jinsha River (27°36’54”N, 99°45’45”E, ~3300 m asl) was excavated in the XB to obtain samples (Fig 1). The top of the section is developed recent soil with massive texture, aggregated structure and abundant plant roots from a depth of 0 to 0.4 m. The section is light grayish green in color and it is mainly composed of silty clay. There are five coarse silt layers in the middle of the section, with thicknesses of 1.7–2.2 m, 3.2–3.5 m, 7–7.2 m, 11.8–12.2 m and 14.2–14.3 m, respectively. The layer between 14.7 and 15.2 m is black clay with enriched carbon. A total of 62 samples were collected from the section at depth intervals of 25 cm for analysis of the geochemical elements. No specific permissions are required for our study locations and sampling activities.

### Analytical methods

The carbonates in the samples were removed from the samples using 1 mol/L HCl before the analysis and were repeated twice. All subsamples consisting of 4 g of each sample (<75 μm) were pressurized with boric acid to 30 t/m^2^ for 20 s. The elemental analysis of the samples was performed using a Philips PW2403 X-ray fluorescence (XRF) spectroscope. The analyzed oxide compounds are identified in elemental form in the Results and Discussion section below. Repeated analyses were performed for every 10 samples to verify the reliability and accuracy of the analytical data. The method has been used to the analysis of standard samples (lake sediment of the national soil standard reference material, GSS-9) with relative standard deviation (RSD) less than 2%.

Seven plant macrofossil samples were collected from the organic-rich horizons of the sediment, and were used to perform ^14^C dating. The ^14^C dates were calibrated to calendar years by the latest calibration program [23]. The XRF analyses and ^14^C dating were performed at the Key Laboratory of Western China's Environmental System of Ministry of Education in Lanzhou University.

## Results and Discussion

### Age model and chronology

**Fig 2:**
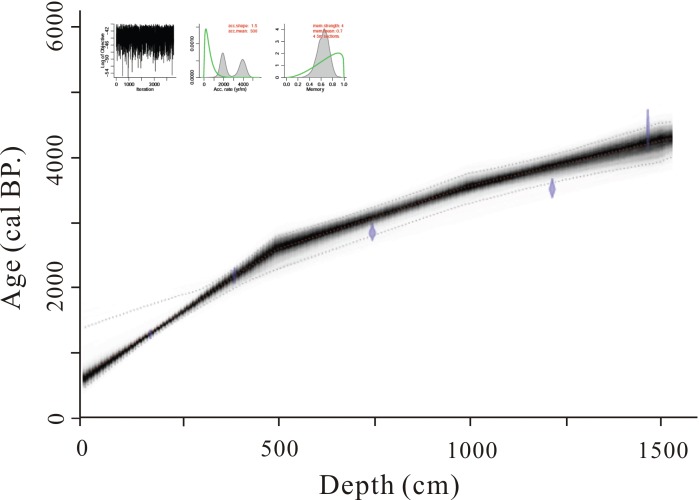
The Bayesian age-depth model of the Xiaozhongdian Basin section.

The measured dates of the study section are presented in Table 1. The ages and the records of the environmental proxies indicate there was continuous sedimentation that extended back to ~42.6 cal ka BP based on the Bayesian model of age-depth (Fig 2). The sedimentation rate in the section is approximately and 0.385 and 0.461 mm/a. The estimated sedimentation rate corresponds with other geochronology in Yunnan Province [24, 25]. The characterties of chemical weathering and climate change in the layer (1.75–0 m) of XB section were not included in the following analyses because it has the only ^14^C data around the Holocene.

**Table 1:** Radiocarbon dating of the lake sediment of Xiaozhongdian Basin and the age model.

Depth (m)	Dating material	AMS ^14^C age (^14^C yr BP)	Calibrated ^14^C (2σ, cal a BP)
1.7	Plant remains	9390–9650	10503–11203
3.9	Plant remains	14505–14915	17425–18421
5.2	Plant remains	16420–16980	19573–20343
7.5	Plant remains	19880–20580	23673–24617
9.4	Plant remains	23380–24370	28215–29360
12.2	Plant remains	25560–26820	30464–31562
14.7	Plant remains	30250–33970	38215–41993

### Geochemical element characteristics

**Fig 3:**
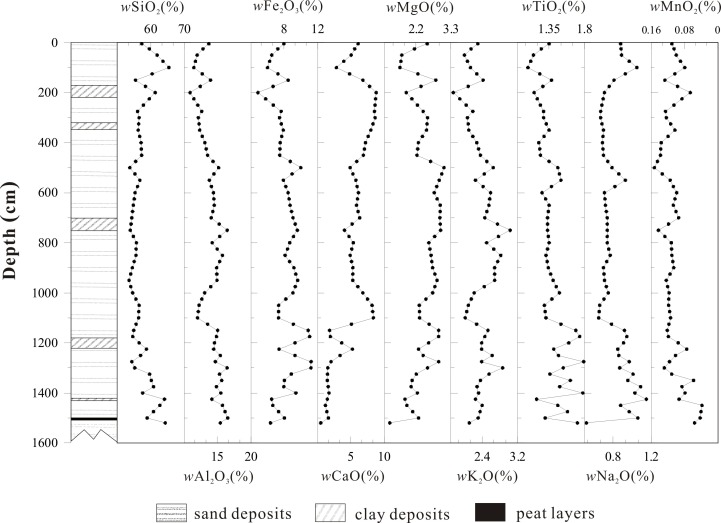
Variation of oxide and element contents in the Xiaozhongdian Basin section.

The compositions of SiO_2_, Al_2_O_3_, Fe_2_O_3_, CaO, MgO, K_2_O, TiO_2_, Na_2_O and MnO_2_ are shown in Fig 3. The total oxide content of the samples ranges from 90.56% to 93.15%, with an average value of 91.76%. SiO_2_ has the highest content of all the analyzed major elements and ranges from 53.51% to 65.40%, with an average value of 57.23%, followed by Al_2_O_3_, with an average content of 14.09%. The regularity of the abundance of oxide compounds in the XB section was as follows: SiO_2_>Al_2_O_3_>Fe_2_O_3_>CaO>MgO>K_2_O>TiO_2_>Na_2_O>MnO_2_. In addition, the content of each element indicated substantial fluctuations for the five sand layers, especially the element Ti, which is generally recognized as being mainly derived from the lake sediments.

### Geochemical proxies and paleoclimatic significance

The characteristics of the geochemical proxies not only indicate the degree of chemical weathering in the lake catchment, but can also indicate the conditions of climate evolution and the changes in the depositional environment [26–28]. Therefore, the geochemical elements of lacustrine deposits can be used to study climate change [29, 30]. The values of (CaO+K_2_O+Na_2_O)/Al_2_O_3_, the index of mineral chemical differentiation (ICV) and the chemical weathering index (CIA) can represent paleoclimatic proxies of the chemical weathering intensity and effective moisture [31, 32].

**Fig 4:**
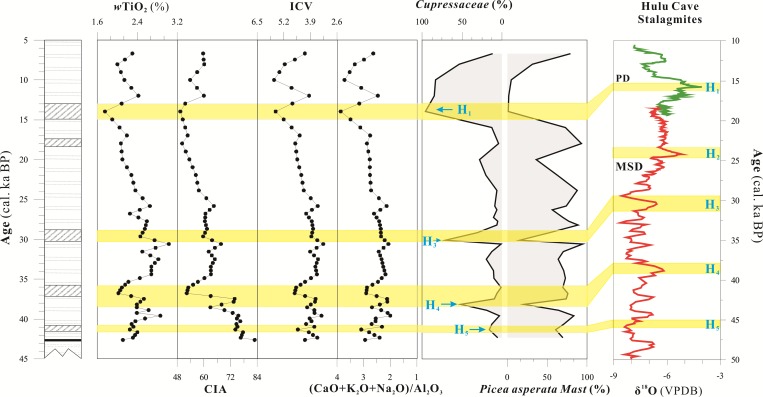
The characteristics of geochemical proxie, sporopollen in the Xiaozhongdian Basin and δ^18^O of Hulu Cave stalagmites [39] (the abundance data for *Cupressaceae* and *Picea asperata Mast* is from [19]).

**Fig 5:**
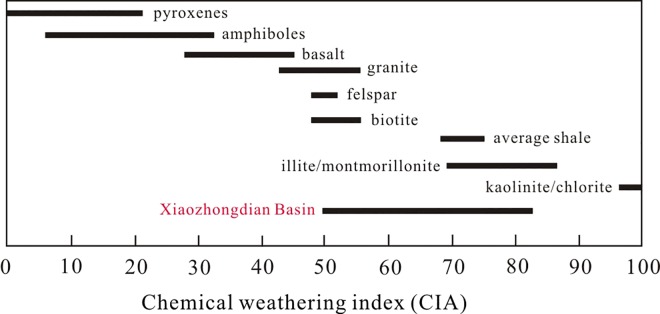
Comparison of CIA for Xiaozhongdian Basin sediments with CIAs for rock and minerals (Based on [33, 34]).

**Fig 6:**
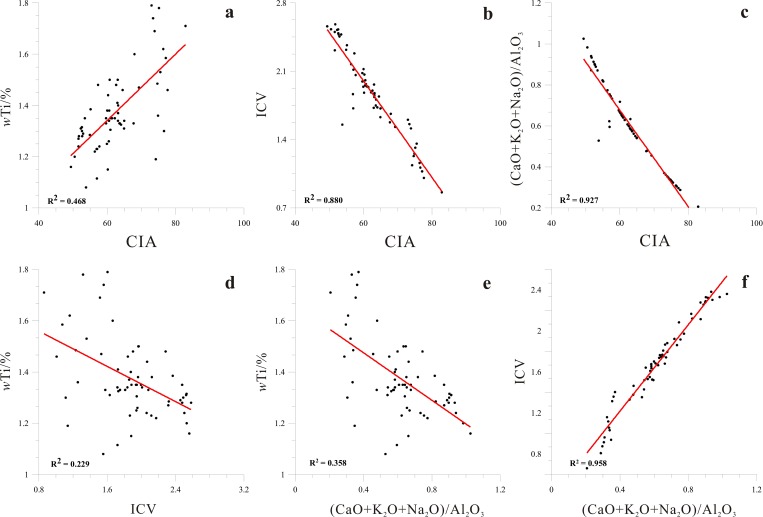
The correlation among the TiO_2_ contents, CIA, ICV and (CaO+K_2_O+Na_2_O)/Al_2_O_3_.

The chemical index of alteration (CIA): The CIA is one of the measures indexes for studying the intensity of chemical weathering [33–35]:

$$\text{CIA}={\text{Al}}_{2}{\text{O}}_{3}/\left({\text{Al}}_{2}{\text{O}}_{3}+{\text{K}}_{2}\text{O}+{\text{Na}}_{2}\text{O}+\text{Ca}O^{*}\right)\times 100$$ (1)

where the oxide amounts are expressed in moles, and CaO* is the amount of CaO in silicates [36–38]. The effect of carbonate minerals has been ruled out in CIA, which mainly reflects the weathering intensity of silicate minerals, so it can well reflect the chemical weathering of the source area. Previous studies have shown that the warm-humid climate and environment would cause to increase the chemical weathering and further analysize the climate of lake catchment by using CIA [29]. The chemical weathering proxies (e.g. CIA, ICV) have been applied to the study of the climate of lake catchment [30–32]. In general, the warm-humid climate and environment would cause to increase the chemical weathering, and CIA values ranging from 70 to 85 indicate the intense chemical weathering and a warm and humid climate. The main source of the lacustrine sediments in Xiaozhongdian paleolake mainly came from the Basin, and it was relatively simple [20]. Therefore, CIA can be seen as the proxy to study the chemical weathering, climatic and environmental change of lake catchment in XB. The CIA of the XB section ranged from 49.37 to 82.90, the major distribution range between 50 and 70, which is the same as the range of CIA values for basalt and average shale throughout the world (Figs 4 and 5), showed that the climatic conditions were weak chemical weathering, and it also indicated the relatively the cold-dry climate, except for the period of 42.6–36.8 cal ka BP (15.2–11.5 m). The CIA varies from 49.4 to 67.7 for the time period of 37.4–11.0 cal ka BP (11.5–1.25 m), with an average value of 58.6, which slightly exceeds the CIA value for feldspar, it indicated the weak chemical weathering and dry climate of the lake catchment. The results of correlation analyses of the proxies indicated a good correlation between the CIA and the content of the element Ti, with R^2^ = 0.468 (P<0.01, Fig 6A).

The index of compositional variability (ICV): Elements preferentially leach and migrate under the warm and humid climate conditions. However, it is difficult for the element Al to migrate during the chemical weathering. Therefore, the ICV can be used to study the proportion of active components in lacustrine deposits. The ICV is expressed as [40]:

$$\text{ICV}=\left({\text{Fe}}_{2}{\text{O}}_{3}+{\text{K}}_{2}\text{O}+{\text{Na}}_{2}\text{O}+\text{CaO}+\text{MgO}+\text{MnO}+\text{Ti}O_{2}\right)/{\text{Al}}_{2}{\text{O}}_{3}$$ (2)

The different minerals have different ICV intervals, the ICV of non-clay minerals is higher than that of clay minerals, and the ICV of pyroxene is between 10 and 100. The ICV values for amphibole, K feldspar, plagioclase, and illite/muscovite are 8, 1, 0.6 and 0.3, respectively, and the range of CIV values for montmorillonite and kaolinite are only 0.15–0.3 and 0.03–0.05, respectively. The high ICV value indicates the intensity of chemical weathering, and reflects the optimal hydrothermal quality for the climatic conditions of the XB [41]. The ICV of the XB ranges from 0.86 to 2.58, with an average of 1.87 (Fig 4), and it is substantially higher than the ICV values of feldspar and clay minerals. The ICV values of XB show an increasing trend from the bottom to the top of the section. The results of the correlation analyses of the proxies indicated good correlations between ICV and Ti and between ICV and CIA, with R^2^ = 0.229 and 0.880, respectively (P<0.01, Fig 6B and 6D). The good correlations indicate the weak chemical weathering and the poor hydrothermal quality of the climatic conditions of the XB at the margin of the SETP.

(CaO+K_2_O+Na_2_O)/Al_2_O_3_: The geochemical behavior of active chemical elements, such as Na, Ca, K, has been affected by climate change during hypergenesis [42]. Aluminum silicate is often changed into clay minerals (e.g. illite, montmorillonite and kaolinite) as a result of chemical weathering. In extremely hot and humid climatic conditions, the clay minerals are decomposed and form bauxite. Therefore, the value of (CaO+K_2_O+Na_2_O)/Al_2_O_3_ in the paleolake in the XB reflect the relationship between the active and inert components and record the change in the climatic conditions. A high value of (CaO+K_2_O+Na_2_O)/Al_2_O_3_ for the lake sediment demonstrate the weak weathering and the low effective moisture dominating the environment and climate. The (CaO+K_2_O+Na_2_O)/Al_2_O_3_ for most of the samples indicates a range from 0.21 to 1.03, with an average value of 0.62 (Fig 4). There are good correlations between (CaO+K_2_O+Na_2_O)/Al_2_O_3_ and Ti, (CaO+K_2_O+Na_2_O)/Al_2_O_3_ and CIA, and (CaO+K_2_O+Na_2_O)/Al_2_O_3_ and ICV (P<0.01, Fig 6C, 6E and 6F). The good correlations demonstrate the weak leaching movement of elements in the section, the low effective moisture and the dry climate of the XB in the Heinrich (H) events.

### Climate evolution and driving mechanism in the SETP

The chemical weathering process and the history of environmental change of the XB between 42.6 and 11 ka cal. BP were reconstructed based on the geochemical characteristics and other climate proxies combined with the regional research [22, 43]. The coarse silt layers in the middle of the section records the multiple extreme climate events of XB, and eventually lead to the disappearance of Xiaozhongdian paleolake. The records of Heinrich events in the lacustrine deposits are particularly noticeable in the section. Previous studies have shown that the records of Heinrich events were difference in the world. The records of Heinrich events were represented as drought in the Bay of Bengal region and India [44, 45]. In the East Asian monsoon region, it appeared as cold and dry [46–49]. However, it indicated the humid climate in South American and the Australian [50, 51]. At the same time, the latest research shows that the signal of stalagmite isotope in China has reflected the dry/wet process under the controlling of monsoon climate [52]. Fig 4 compares the distribution patterns between the geochemical records of the lacustrine deposits and δ^18^O of Hulu Cave stalagmite [39]. By comparison, Heinrich events were recorded relatively well in the geochemical and sporopollen records of XB and δ^18^O of Hulu Cave stalagmite, except H_2_ event. The records of H_1_, H_3_, H_4_ events are relatively more remarkable, and that of H_5_ less obvious. In general, the instabilities of the climatic characteristics of the SWAM are mainly affected by the interaction among ice sheets, the ocean and the atmosphere and the climate signals of high latitudes passing to low latitudes through thermohaline circulation [53]. The TP can be regarded as a regulator that enlarged or reduced the signals during the H events in the SETP [54]. During the H events, the cold signals were transmitted from the high latitudes of the North Atlantic to the TP. In turn, this effect caused the cooling effect to be very strong on the TP as a result of the upper-level westerly jet stream and then reduced the suction action associated with the SWAM [55], thus accelerated the drying rate of Xiaozhongdian Basin, which was amplifying the degree of drought in Heinrich events.

## Conclusions

The geochemical characteristics and their parameters for the Xiaozhongdian Basin lacustrine deposits recorded the millennial scale history of the SWAM evolution and a series of the drought events corresponding to the Heinrich events.

By comparing research conducted in the study region and other regions, the TP can be considered a climate regulator that enlarged or reduced the signals associated with the suction action during the H and D/O events. The forms of the climatic events in the various regions of China are related to the effect of the suction action of the TP. The record of the Heinrich events of the SETP indicated more substantial drought events. The warmer climate further increased the evaporation of the study area, which was amplifying the degree of drought. However, the regional records for other regions of China have indicated rapid cooling because of the less pronounced effect by the TP. It would be beneficial to explore the regional climate characteristics and their relationships with the global patterns.

## References

[1] Taylor SR, McLennan SM. The Continental Crust: its Composition and Evolution London: Blackwell scientific publications 1985.

[2] Zhang HC, Zhang WX, Chang FQ, Yang LQ, Lei GL, Yang MS, et al Geochemical fractionation of rare earth elements in lacustrine deposits from Qaidam Basin. Science in China 2009; 52: 1703–1713.

[3] Li MH, Zhu LP, Wang JB, Wang LQ, Yi CL, Galy A. Multiple implications of rare earth elements for Holocene environmental changes in Nam Co, Tibet. Quaternary International 2011; 236: 96–106.

[4] Overpeck J, Anderson D, Trumbore S, Prell W. The southwest Indian Monsoon over the last 18,000 years. Climate Dynamics 1996; 12(3): 213–225.

[5] Sahai AK, Grimm AM, Satyan V, Pant GB. Long-lead prediction of Indian summer monsoon rainfall from global SST evolution. Climate Dynamics 2003; 20(7–8): 855–863.

[6] Tarasov P, Granoszewski W, Bezrukova E, Brewer S, Nita M, Abzaeva A, et al Quantitative reconstruction of the last interglacial vegetation and climate based on the pollen record from Lake Baikal, Russia. Climate Dynamics 2005; 25(6): 625–637.

[7] Wang PX, Clemens S, Beaufort L, Braconnot P, Ganssen G, Jian Z, et al Evolution and variability of the Asian monsoon system: state of the art and outstanding issues. Quaternary Science Reviews 2006; 24(5–6): 595–629.

[8] Vogel H, Wagner B, Zanchetta G, Sulpizio R, Rosén P. A paleoclimate record with tephrochronological age control for the last glacial-interglacial cycle from Lake Ohrid, Albania and Macedonia. Journal of Paleolimnology 2010; 44(1): 295–310.

[9] Bartlein PJ, Harrison SP, Brewer S, Connor S, Davis BAS, Gajewski K, et al Pollen-based continental climate reconstructions at 6 and 21 ka: a global synthesis. Climate Dynamics 2011; 37(3–4): 775–802.

[10] Xu H, Hong Y, Hong B. Decreasing Asian summer monsoon intensity after 1860 AD in the global warming epoch. Climate Dynamics 2012; 39(7–8): 2079–2088.

[11] Zhang WX, Shi ZT, Chen GJ, Liu Y, Niu J, Ming QZ, et al Geochemical characteristics and environmental significance of Talede loess-paleosol sequences of Ili Basin in Central Asia. Environmental Earth Sciences 2013; 70(5): 2191–2202.

[12] Clark MK, Schoenbohm LM, Royden LH, Whipple KX, Burchfiel BC, Zhang X, et al Surface uplift, tectonics, and erosion of eastern Tibet from large-scale drainage patterns. Tectonics 2004; 23(1): 241–262.

[13] Zhu LP, Lü XM, Wang JB, Peng P, Kasper T, Daut G, et al Climate change on the Tibetan Plateau in response to shifting atmospheric circulation since the LGM. Scientific Reports 2015; 5: 13318 10.1038/srep13318 26294226 PMC4543934

[14] Li J, Fang X. Uplift of the Tibetan Plateau and environmental changes. Chinese Science Bulletin 1999; 44(23): 2117–2124.

[15] Kong P, Zheng Y, Caffee MW. Provenance and time constraints on the formation of the first bend of the Yangtze River. Geochemistry Geophysics Geosystems 2012; 13(6): 96–109.

[16] Cook ER, Anchukaitis KJ, Buckley BM, D'Arrigo RD, Jacoby GC, Wright WE. Asian monsoon failure and megadrought during the last millennium. Science 2010; 328: 486–489. 10.1126/science.1185188 20413498

[17] Ponton C, Giosan L, Eglinton TI, Fuller DQ, Johnson JE, Kumar P, et al Holocene aridification of India. Geophysical Research Letters 2012; 39(3): L03704.

[18] Sinha A, Stott L, Berkelhammer M, Cheng H, Edwards RL, Buckley B, et al A global context for megadroughts in monsoon Asia during the past millennium. Quatuernary Science Review 2011; 30: 47–62.

[19] Ming QZ, Su H, Shi ZT, Dong M, Zhang WX. Last Five Heinrich Events Revealed by Lacustrine Sediments from Xiaozhongdian Basin in Yunnan Province. Acta Geographica Sinica 2011; 66(1): 123–130.

[20] Zhao XT, Zheng MP, Li DM. Formation and Evolution of the Ancient "Lake Xiaozhongdian" in Diqing, Yunnan and Its Relationship with Development of the Ancient "Lake Shigu" and the Modern Valley of the Jinsha River. Acta Geologica Sinica 2007; 81(12): 1645–1651.

[21] Xu H, Sheng E, Lan J, Liu B, Yu K, Che S, et al Decadal/multi-decadal temperature discrepancies along the eastern margin of the Tibetan Plateau. Quaternary Science Reviews 2014; 89: 85–93.

[22] Yin Y, Fang NQ, Sheng JF, Hu CY, Nie HG. Lacustrine records of environmental changes during the last 57 ka in the Napahai lake, northwestern Yunnan, China. Marine Geology & Quaternary Geology 2002; 22(4): 99–105.

[23] Reimer PJ, Bard E, Bayliss A, Beck JW, Blackwell PG, Ramsey CB, et al IntCal13 and Marine13 radiocarbon age calibration curves 0–50,000 years cal BP. Radiocarbon 2013; 55(4): 1869–1887.

[24] Hodell DA, Brenner M, Kanfoush SL, Curtis JH, Stoner JS, Song X, et al Paleoclimate of southwestern China for the past 50,000 yr. inferred from lake sediment records. Quaternary Research. 1999; 52: 369–380.

[25] Zhang WX, Ming QZ, Shi ZT, Chen GJ, Niu J, Lei GL, et al Lake sediment records on climate change and human activities in the Xingyun Lake catchment, SW China. Plos One 2014; 9(3): e02167. 10.1371/journal.pone.0102167 PMC4102491 25033404

[26] Mclennan S M. Weathering and global denudation. Journal of Geology. 1993; 101: 295–303.

[27] Liu L, Wang H, Chen J. Reconstruction of the rainfall on the Chinese Loess Plateau during the past 130 ka from the dolomite distributions. Geochim Cosmochim Acta 2006; 70: 363.

[28] Li Y, Wang N, Cheng HY, Long H, Zhao Q. Holocene environmental change in the marginal area of the Asian monsoon: a record from Zhuye Lake, NW China. Boreas 2009; 38(2): 349–361.

[29] Roy PD, Caballero M, Lozano S, Morton O, Lozano R, Jonathan MP, et al Provenance of sediments deposited at paleolake San Felipe, western Sonora Desert: Implications to regimes of summer and winter precipitation during last 50 cal kyr BP. Journal of Arid Environments 2012; 81: 47–58.

[30] Park J, Lim HS, Lim J, Park YH. High-resolution multi-proxy evidence for millennial- and centennial-scale climate oscillations during the last deglaciation in Jeju Island, South Korea. Quaternary Science Reviews 2014; 105: 112–125.

[31] Lee MK, Lee YI, Lim HS, Lee JI, Yoon HI. Late Pleistocene-Holocene records from Lake Ulaan, southern Mongolia: implications for east Asian palaeomonsoonal climate changes. Journal of Quaternary Science 2013; 28(4): 370–378.

[32] Wu YH, Li SJ, Xia WL. Element geochemistry of lake sediment from Gourenco lake, Kekexili, Qinghai-Xizang plateau and its significance for climate variation. Journal of Earth Science & Enivronmental 2004; 26: 64–68.

[33] Nesbitt HW, Young GM, Mclennan SM, Keays RR. Effects of Chemical Weathering and Sorting on the Petrogenesis of Siliciclastic Sediments, with Implications for Provenance Studies. Journal of Geology 1996; 104(5): 525–542.

[34] Young GM. Geochemical investigation of a Neoproterozoic glacial unit: the mineral fork formation in the Wasatch Range. Utah. GSA Bull 2002; 114: 387–399.

[35] Wei ZQ, Zhong W, Chen YQ, Tan LL. Supergene geochemical elements of swampy basin in the subtropical monsoon region: a case study of Dingnan Dahu in Jiangxi Province. Progress in Geography 2015, 34: 909–917.

[36] Nesbitt HW, Markovics G, Price RC. Chemical processes affecting alkalis and alkaline earths during continental weathering. Geochim Cosmochim Acta 1980; 44: 1659–1666.

[37] Nesbitt HW, Young GM. Early Proterozoic climates and plate motions inferred from major element chemistry of lutites. Nature 1982; 299: 715–717.

[38] Gallet S, Jahn BM, Lanoë BVV, Dia A, Rossello E. Loess geochemistry and its implications for particle origin and composition of the upper continental crust. Earth & Planetary Science Letters 1998; 156(3–4): 157–172.

[39] Wang YJ, Cheng H, Edwards RL, An ZS, Wu JY, Shen CC, et al A high-resolution absolute-dated late Pleistocene Monsoon record from Hulu Cave, China. Science 2002; 294(5550): 2345–2348. 10.1126/science.1064618 11743199

[40] Cox R. The influence of sediment recycling and basement composition on evolution of mudrock chemistry in the southwestern United States. Gechimica et Cosmochimica Acta 1995; 59: 2919–2940.

[41] Joo YJ, Lee YI, Bai Z. Provenance of the Qingshuijian Formation (Late Carboniferous), NE China: Implications for tectonic processes in the northern margin of the North China block. Sedimentary Geology 2005; 177(1): 97–114.

[42] Zhang WX, Zhang HC, Lei GL, Yang LQ, Niu J, Chang FQ, et al Elemental geochemistry and paleoenvironment evolution of Shell Bar section at Qarhan in the Qaidam Basin. Quaternary Sciences 2008; 28: 917–928.

[43] An ZS, Clemens SC, Shen J, Qiang XK, Jin ZD, Sun YB, et al Glacial-interglacial indian summer monsoon dynamics. Science 2011; 333: 719–23. 10.1126/science.1203752 21817044

[44] Peterson LC, Haug GH, Hughen KA, Röhl U. Rapid changes in the hydrologic cycle of the tropical Atlantic during the last glacial. Science 2000; 290: 1947–1951. 11110658 10.1126/science.290.5498.1947

[45] Stott L, Poulsen C, Lund S. Super ENSO and global climate oscillations at millennial time scales. Science 2002; 297: 222 10.1126/science.1071627 12114618

[46] Yuan DX, Cheng H, Lawrence ER, Dykoski CA, Kelly MJ, Zhang M, et al Timing, Duration, and Transitions of the Last Interglacial Asian Monsoon. Science 2004; 304(5670): 575–578. 10.1126/science.1091220 15105497

[47] Dykoski CA, Edwards RL, Cheng H, Yuan DX, Cai YJ, Zhang M, et al A high-resolution, absolute-dated Holocene and deglacial Asian monsoon record from Dongge Cave, China. Earth & Planetary Science Letters 2005; 233(s 1–2): 71–86.

[48] Wang YJ, Cheng H, Edwards RL, Kong XG, Shao XH, Chen ST, et al Millennial- and orbital-scale changes in the East Asian monsoon over the past 224,000 years. Nature 2008; 451(7182): 1090–1093. 10.1038/nature06692 18305541

[49] Zhou H, Zhao JX, Feng Y, Chen Q, Mi X, Shen CC, et al Heinrich event 4 and Dansgaard/Oeschger events 5–10 recorded by high-resolution speleothem oxygen isotope data from central China. Quaternary Research 2014; 82(2): 394–404.

[50] Leuschner DC, Sirocko F. The low-latitude monsoon climate during Dansgaard-Oeschger cycles and Heinrich events. Quaternary Science Reviews 2000; 19(1–5): 243–254.

[51] Muller J, Kylander M, Wüst RAJ, Weiss D, Martinez-Cortizas A, Legrande AN, et al Possible evidence for wet Heinrich phases in tropical NE Australia: The Lynch's Crater deposit. Quaternary Science Reviews 2008; 27(5–6): 468–475.

[52] Wang YJ, Liu DB. Speleothem records of Asian paleomonsoon variability and mechanisms. Chinese Science Bulletin 2016; 61: 938–951.

[53] Rahmstorf S. Rapid climate transitions in a coupled ocean-atmosphere model. Nature 1994; 372: 82–85.

[54] Fang XM, Lü LQ, Mason JA, Yang SL, An ZS, Li J, et al Pedogenic response to millennial summer monsoon enhancements on the Tibetan Plateau. Quaternary International 2003; 2(106–107): 79–88.

[55] Weber ME, Wiedicke-Hombach M, Kudrass HR, Erlenkeuser H. Bengal Fan sediment transport activity and response to climate forcing inferred from sediment physical properties. Sedimentary Geology 2003; 155(3–4): 361–381.

